# Personal.—Mississippi Valley Dental Society

**Published:** 1895-05

**Authors:** 


### Personal.—Mississippi Valley Dental Society.

Drs. Wright and Hunter are open for engagements as inductors of Presidents or indeed almost any thing else. An inimitable combination.

Dr. Geo. W. Evans, of N. Y., to the great gratification of all was present, and loaded to the muzzle with good things, which he liberally dispensed to the great edification of all present; and for which he received a most hearty vote of thanks. He presented much of detail and minutiæ, in regard to crown and bridge-work, which is greatly needed to obtain the best results in this department of practice. "Evans on Crown and Bridge-work," is a most excellent work, indeed; but it is not equal to the author for practical points.

Drs. Harlan and Eams of Chicago were present, and did much to add to the profit and interest of the occasion. They emphatically belong to that number who are always heartily welcomed to all Dental Associations, for they are ever ready to contribute to every good work, their effort for the advacement of the profession.

Dr. J. E. Cravens, of Indianapolis, was present and ready as usual for any work that might be asked of him, for the promotion of professional interest. He is always loaded for Pyorrhœa Alveolaris, and prompt root filling.

Drs. Brown and Mason were welcome guests and added interest to the occasion.

Dr. Morrell, of New Albany, was present and made glad his old friends and especially those who have known him from long ago as a worker in Association effort.

